# Dynamic evolution of locomotor performance independent of changes in extended phenotype use in spiders

**DOI:** 10.1098/rspb.2023.2035

**Published:** 2023-10-25

**Authors:** Michael B. J. Kelly, Md Kawsar Khan, Kaja Wierucka, Braxton R. Jones, Ryan Shofner, Shahan Derkarabetian, Jonas O. Wolff

**Affiliations:** ^1^ Evolutionary Biomechanics, Zoological Institute and Museum, University of Greifswald, Loitzer Strasse 26, 17489 Greifswald, Germany; ^2^ School of Natural Sciences, Macquarie University, Sydney, New South Wales 2109, Australia; ^3^ Institute of Biology, Freie Universität Berlin, Königin-Luise-Straße 1-3, 14195 Berlin, Germany; ^4^ Behavioural Ecology and Sociobiology Unit, German Primate Center - Leibniz Institute for Primate Research, Kellnerweg 4, 37077 Göttingen, Germany; ^5^ School of Biological Sciences, University of Sydney, Camperdown, New South Wales 2006, Australia; ^6^ Evolution and Ecology Research Centre, School of Biological, Earth and Environmental Sciences E26, University of New South Wales, Sydney 2052, Australia; ^7^ Department of Organismic and Evolutionary Biology, Museum of Comparative Zoology, Harvard University, Cambridge, MA 02138, USA

**Keywords:** animal performance, extended phenotype, spider web, prey capture, Desidae

## Abstract

Many animals use self-built structures (extended phenotypes) to enhance body functions, such as thermoregulation, prey capture or defence. Yet, it is unclear whether the evolution of animal constructions supplements or substitutes body functions—with disparate feedbacks on trait evolution. Here, using brown spiders (Araneae: marronoid clade), we explored if the evolutionary loss and gain of silken webs as extended prey capture devices correlates with alterations in traits known to play an important role in predatory strikes—locomotor performance (sprint speed) and leg spination (expression of capture spines on front legs). We found that in this group high locomotor performance, with running speeds of over 100 body lengths per second, evolved repeatedly—both in web-building and cursorial spiders. There was no correlation with running speed, and leg spination only poorly correlated, relative to the use of extended phenotypes, indicating that web use does not reduce selective pressures on body functions involved in prey capture and defence *per se*. Consequently, extended prey capture devices serve as supplements rather than substitutions to body traits and may only be beneficial in conjunction with certain life-history traits, possibly explaining the rare evolution and repeated loss of trapping strategies in predatory animals.

## 1. Introduction

Predators rely on behavioural, physiological and morphological adaptations to successfully capture and subdue prey. The ability to move fast is a key trait of many predatory strategies, but it is also energetically costly, and should thus be under strict selective pressure [1,2]. Some predators alternatively invest in the production of adhesive secretions or snares that intercept and immobilize prey without the requirement of quick muscular action [3]. Such examples comprise the silken webs of spiders and glowworms, the projectile slime of velvet worms or ant-lion pits [3–5]. The pathways and conditions leading to the evolution of such external devices—extended phenotypes [6]—and how they interactively evolve with body traits is poorly understood [7,8]. Trap building is an overall rare strategy among predatory animals and has been lost repeatedly subsequent to its evolution [5,8]. Consequently, high direct (e.g. investments in construction) and indirect costs (e.g. increased exposure to predators), and limitations in efficacy have been hypothesized for the use of external prey capture devices [5,9,10]. However, thus far, empirical and cross-species approaches are lacking in order to identify the true costs, ecological limitations and evolutionary dynamics of trap-based predation [5].

Extended phenotypes, such as spider webs, could reduce the need to maintain costly morphological and physiological adaptations to functions (such as prey interception and immobilization) that are thereby rendered redundant (*substitution*). As a consequence, such traits would show decreased levels of expression after the evolution of extended phenotypes, and increased levels of expression if extended phenotypes get lost again in a lineage. As the production of extended phenotypes brings its own costs, substitution can only be successful if the costs of maintaining the substituted body traits are higher. By contrast, the extended phenotype could serve as an additional supplement (or complementation) to the body function, but does not lower selective pressures to maintain a high expression of traits related to this function in the body, e.g. because the extended phenotype function works only in conjunction with the primary body trait performing as efficient as in its absence (*supplementation*). For instance, silk lines that serve as an extension of the sensory system by transmitting vibratory information from distantly moving prey to the spider still require the possession of vibration sensors and signal processing systems [11,12]. Here, the extended phenotype adds to the function and may aid in overcoming limits in the evolvability of the primary body function. As in the case of supplementation both primary and extended traits are expressed in concert, the overall costs towards predatory functions may be increased, and hence, only pay off if resulting in a higher prey yield (e.g. larger or more prey can be caught and consumed than in the absence of an extended prey capture device).

If predators do not use snares, but hunt down and subdue prey with a strike, speed is not enough, but further morphological features such as teeth or claws are required to stop and hold the prey. Some spiders—including many marronoids—bear a double row of long, stiff hydraulic spines (macrosetae) on their front legs. These spines become erect during the rapid predatory strike with the legs grasping the prey, where they form a barrier (capture basket) to prevent prey from escaping between the legs, before being immobilized with the fangs and venom [13]. After the attack, the spines fold back into their original position, lying flat on the leg's cuticle. This suggests a primary function of these spines in prey capture and was therefore chosen as an example of morphological adaptation to prey capture.

Here, we tested if the evolution of physiological (sprint speed) and morphological traits (leg spination) correlates with predatory strategy: the striking of prey versus the trapping of prey with a web. These two traits were chosen as they: (i) are comparably easy to quantify, (ii) are considered to have a strong effect on active prey capture efficacy [13] and (iii) because both traits exhibit a high interspecific variability within the focal group including reports of remarkably high expression in some species [14,15]. We focused on a clade of spiders that exhibits multiple web losses and gains [8,16,17] (representing evolutionary replicates), the so-called marronoid (brown) clade of spiders (Araneae: Amaurobioidea). The marronoid clade belongs to the large group of araneomorph spiders and contains nine poorly defined families with unstable taxonomy, including widely known species such as the hobo spider (Agelenidae: *Eratigena agrestis*), the grey house spider (Desidae: *Badumna longinqua*) and the water spider (Dictynidae: *Argyroneta aquatica*) [18]. One of the reasons for the taxonomic instability is the phenotypic and ecological diversity with many homoplastic traits observed in this group, which makes it hard to determine diagnostic characters, but renders the marronoid spiders highly suitable for comparative studies of trait evolution.

We hypothesized that: (i) sprint speed and leg spination are less expressed in web-building than in non-web-building species (prediction of the ‘*substitution' hypothesis*), or (ii) there is no such difference, or sprint speed and leg spination are more expressed in web-building than in non-web-building species (prediction of the ‘*supplementation' hypothesis*). As webs may differ in their efficacy to immobilize prey, we included a test of the substitution hypothesis related to the capability to produce costly but efficient adhesive capture threads (so-called ‘cribellar') versus such species that are incapable of producing such sticky threads (so-called ‘ecribellar’), which may include both web-building and non-web-building species. As an additional test, if our focal traits are affected by habitat structure, we compared trait expression between primary ground-dwelling and above-ground-dwelling species.

## 2. Material and methods

### (a) Animal collection and material sourcing

Spiders were collected in New South Wales, South Queensland, Tasmania, the South Island of New Zealand and in Germany under scientific licenses SL101868, FA18285, PTU19-001938 and 71225-RES. Tissue samples and specimens for morphology for some species were sourced from museum and institutional collections. Species were identified with primary or (if available) secondary taxonomic literature. In addition, in some cases, specimens were compared with type specimens for taxonomic identification. Vouchers were preserved in ethanol and deposited at curated arachnological collections. The full list of specimens used in the phylogenomic study, including their collection data and voucher locations are found in electronic supplementary material, S1 and S2.

During field collections and housing the spiders in captivity, notes of the microhabitat, the presence of a web and details of the web or retreat (if present) were recorded and photo-documented where possible.

The coding of ecological traits followed our field observations and a literature survey (details in electronic supplementary material, S7 and S8).

### (b) Video recording and tracking analysis

Videos were captured with a BASLER Ace camera (640 × 480 pixels, 750 fps, 1/4’ CMOS Monochrome) equipped with a Fujinon HF12.5HA-1B lens (F1.4 - F16, 12.5 mm) and 0.5–40 mm extension tubes using the TroublePix software, or with a Phantom Miro high speed video camera equipped with a Canon DSLR lens. Videos were taken at 100–500 frames per second (depending on the base speed of the spider). Adult males were not included in the study as they often have significantly longer legs and smaller bodies and a different locomotor ecology than female and juvenile spiders. Spiders missing any of their legs were omitted from the analysis.

Running speed of spiders was recorded in the laboratory or fieldwork accommodation at room temperature. Spiders ran either on a polished timber bar (50 cm long, 10 cm wide) enclosed with acrylic glass sheets, or on a paper sheet in a polypropylene box (30 × 20 cm). Spiders were released from one end of the running track and their movement filmed from vertically above. If the spider did not run, or only walked at slow speed, it was touched on the posterior portion of the abdomen to trigger an escape response. Unless the spider showed fatigue, running trials were repeated three to five times. Each video contained a reference centimetre scale in the field of view.

From each video, the total body length of the spider was measured (from the front of the cephalothorax to the end of the abdomen). We then inspected the paths of the spiders and included only those where spiders ran in a constant direction in the analyses.

Using the plugin *MTrackJ* [19] in *ImageJ* [20], spiders were tracked in the video frame by frame (using the anterior edge of the abdomen as a reference point). The resulting series of *x*-*y* coordinates were then exported as a csv file and further processed in R 4.0.1 [21] using automated scripts (electronic supplementary material, S3). The distance travelled between frames was converted from pixels into centimetres (using the reference scale present in the video frame) and the velocity calculated between frames (from distance travelled and frame rate of the recording). The per frame pair velocity values for each recording were smoothed with the function *smooth.spline* with the number of knots assigned to *N*/2 + 1, where *N* is the number of measured datapoints (frames) in the video. This was done to remove the typical oscillations of the body centre to estimate the velocity of the whole animal. Then the mean speed and sprint speed (maximum after smoothing) was calculated both absolute (in cm s^−1^) and relative (in body lengths per second, bl/s). For the comparative analysis, the maximal value of the sprint speed among all trials was selected for each individual and the mean of these values for all individuals was calculated for each species.

### (c) Morphometric measurements

Ethanol-preserved specimens were photographed in 70–80% ethanol on a Zeiss Discovery.V20 (inserting the automatically calculated scale bars) or with a Canon DLSR on a Motic stereo microscope (including photos of a micrometre scale). The body was photographed from dorsal and lateral angles. Front and hind legs were removed on one side and their prolateral side was photographed.

Measurements (in millimetres) were performed in *ImageJ*. Body length was measured from the front edge of the carapace to the posterior end of the abdomen (without spinnerets). Carapace width was measured at the widest point. Leg segments were measured between condyles excluding the coxa, trochanter and pretarsus. The spines (macrosetae), fully visible from the prolateral side (i.e. including the base socket), were counted on all measured segments of the front leg and the sum of the length of all these spines (from the base socket to the tip) was calculated. This sum was divided by the sum of the length of all measured leg segments giving the spination index. In ethanol-preserved material, it is not possible to distinguish which spines are hydraulic; therefore, we included all spines, including lateral and dorsal spines that are permanently erect. Spines are distinguishable from other setae by their strong sclerotization (often black or dark brown colour), straight shaft, thick base socket and the absence of microtrichia. The relative leg length was calculated as the sum of all measured segments of the posterior leg divided by carapace width.

### (d) DNA extraction and analysis of ultraconserved elements

Genomic DNA extraction of all samples was performed using either the leg(s) or the whole specimen (dependent on the size of the spider), following the DNeasy Blood and Tissue Kit (Qiagen, Valencia, CA, USA) manufacturer's protocol and quantified using a Qubit fluorometer (Life Technologies, Inc.). Ultraconserved elements (UCE) library preparations were performed following the protocol of Starrett *et al*. [22] and Derkarabetian *et al*. [23], as well as the Hybridization Capture for Targeted next generation sequencing (NGS) manual v4.01 protocol (https://arborbiosci.com/wp-content/uploads/2018/04/myBaits-Manual-v4.pdf). Library preparation for a subset of the samples (*n* = 23) was conducted using the MYbaits Arachnida 1.1Kv1 kit (Arbor Biosciences, Ann Arbor, MI, USA) [24] (see details in electronic supplementary material, S1) and sequenced on a NovaSeq 6000 at the Bauer Core Facility at Harvard University. For the remaining samples (*n* = 75), the extracted DNA was dried using an Eppendorf Concentrator plus speed-vac and transported to NGS Division, Arbor Biosciences (Ann Arbor, MI, USA) for UCE library preparation using the Spider 2Kv1 kit [25].

Processing of the raw demultiplexed read data was performed using the PHYLUCE v1.6.8 pipeline [26]. Reads were cleaned with the Trimmomatic wrapper [27], Illumiprocessor [28], using default settings, and then assembled using both Trinity v2.1.1 [29], with default settings, and ABySS v1.5.2 [30] (using the 64-kmer value setting), and the results combined into a single assembly file. Probes were matched to contigs using the Spider 2Kv1 probeset file using minimum coverage and minimum identity values of 65. The UCE loci were aligned using MAFFT [31] and trimmed using GBLOCKS [32,33] with custom gblocks settings (b1 = 0.5, b2 = 0.5, b3 = 6, b4 = 6) applied in the PHYLUCE pipeline. Aligned UCEs were then imported into Geneious 11.1.5 [34] and visually inspected for obvious alignment or sequencing errors.

### (e) Phylogenetic analysis

Phylogenetic analyses of the final matrix were performed using two phylogenetic inference methods: Maximum Likelihood (ML) and Bayesian inference (BI). The ML analysis was conducted using IQ-TREE v2.1.3 [35], implementing ModelFinder [36], to estimate the best-fit partitioned models by locus [37]. The ultrafast bootstrap technique with 1000 replicates was used to quantify the support of phylogenetic relationships [38].

The final matrix was further trimmed with the more conservative gblocks settings (b1 = 0.5, b2 = 0.85, b3 = 4, b4 = 8) prior to Bayesian analysis. To make the BI computationally feasible, the UCE dataset was reduced by subsampling the most informative loci [39]. Gene trees were inferred with ParGenes v. 1.0.1 [40], with optimal models selected according to Bayesian information criterion (BIC), and 100 bootstrap replicates. Gene selection was made with the script of Mongiardino Koch [39], specifying minimum occupancy of 50% and discarding 5% of outlier genes. BI was performed using BEAST 2.0 [41] with GTR + G substitution model, Relaxed clock lognormal and a birth–death tree model. To time-calibrate the tree, lognormal distributed age priors were placed to some nodes, informed by the age of two fossils (*Eohahnia succini* Petrunkevitch and *Vectaraneus yulei* Selden) and five secondary calibration points taken from Magalhães *et al*. [42]. One analysis was run without monophyly constraints, and a second analysis constraining the Nearctic Agelenidae *sensu stricto* to the base of all other marronoids (except *Amaurobius*). Four independent runs of 200 million generations were run for each dataset. The first 30% of each run was dropped as burn-in before building the consensus tree using the *TreeAnnotator* app of the BEAST package.

The topology of the phylogenies produced by the ML and BI analyses were then visualized and compared using *FigTree* v1.4.3.

### (f) Comparative analysis

The following terminals were dropped for the comparative analysis due to a lack of trait data (because only male material was available): Matachiinae spec. 4 and *Nuisiana arboris*. Further, species for which trait data but no phylogenetic information was available were not included in the phylogenetic comparative analysis. Analyses were repeated using two alternative topologies (unconstrained BEAST tree, and BEAST tree where Agelenidae was constrained to an early diverging node as found in the ML analyses).

The evolution of web-building behaviour was inferred using the stochastic character mapping approach implemented in the *R* package *phytools* [43]. Three alternative evolutionary models were considered: (1) ER, equal rates (i.e. web loss and gain occur at same rates), (2) ARD, all rates different (web loss and gain occur at unequal rates), (3) customized model where web re-evolution is suppressed (Dollo's Law). Model fit was compared using corrected Akaike information criterion (AICc) weights.

For continuous traits (sprint speed and spination index), the following models were fitted using the package *geiger* 2.0 [44]: (1) BM, Brownian Motion, (2) OU, Ornstein–Uhlenbeck model, (3) EB, Early Burst model, (4) *λ*, Pagel's lambda. Trait evolution was plotted with the *contMap* function in *phytools*.

The expression of continuous traits were compared between ecological categories (web builders versus cursorial spiders; cribellar versus ecribellar; ground dwelling versus inhabiting above-ground microhabitats) with phylogenetic linear regressions in the R package *phylolm* [45] and branch length transformations based on the best fitting model (*λ* for running speed and OU for spination). Effect sizes were estimated using *DurgaDiff* function with 5000 bootstrap replicates and effect size plots were generated using *DurgaPlot* function of the *Durga* R package [46].

Scripts and input files for the comparative analyses are found in the electronic supplementary material, S4.

## 3. Results

### (a) UCE sequencing and phylogenetic results

Sequenced samples contained an average of 4 072 740 reads per sample (post trimming; s.d. ± = 2 210 776) and an average of 257 754 contigs (s.d. ± = 226 935). The final matrix (electronic supplementary material, S5) included 1266 UCE loci, produced from the assembled contigs across all taxa, with an average of 929 loci per sample (s.d. ± = 381; electronic supplementary material, S1). The number of UCE loci obtained for taxa processed using the Arachnida 1.1Kv1 kit ranged between 181 and 555 with an average of 251 UCEs per sample (s.d. ± = 79). Those taxa processed using the Spider 2Kv1 kit produced UCE loci ranging from 950 to 1215 with an average of 1137 UCEs per sample (s.d. ± = 43).

**Figure 1.**
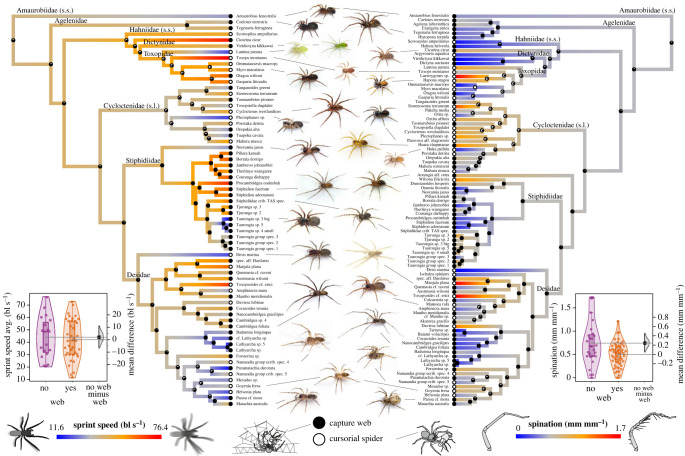
Macroevolution of locomotor performance, capture spines and predatory traps in the marronoid clade of spiders. Coloured Bayesian phylogenies based on BEAST analysis with fixed position of Agelenidae (note that the systematics of the marronoid clade is due for formal revision and indicated family delimitations are tentative); colours indicate trait levels (see respective legend below), circles at tips indicate the species' foraging mode (see legend in middle below; for further details, see electronic supplementary material, S8), and circles at nodes indicate the posterior probability of web use in the most recent common ancestor (assuming equal rates of web loss and gain). Inserted box and violin effect size plots indicate differences in trait means between web builders and cursorial hunters. Boxplots display the group median and the 75th and 25th percentiles and whiskers extend to the minimum and maximum but exclude outliers that are beyond 1.5 times the interquartile range and the dots indicating the individual species means. Half violin in the effect size plots exhibit the distribution of bootstrapped differences; the solid square shows mean difference, while the vertical bar shows 95% confidence interval of mean difference. bl/s = body lengths per second.

Phylogenetic inference produced trees with overall high node supports (i.e. ubf-values greater than 95 for 93 of 97 nodes, electronic supplementary material, S6). Node support dropped slightly when applying strict gblock settings (i.e. removing much of the variable sequence regions) (ubf-values greater than 95 for 90 of 97 nodes, electronic supplementary material, S6). There was one major discordance in the topology between ML and BI trees, with a different position of the Nearctic Agelenidae *sensu stricto*. Both topologies have been found in previous phylogenomic studies [47,48] and therefore, we ran our comparative analyses on both alternative phylogenies. Figure 1 shows the topology found by the BI analysis with Agelenidae fixed to the base of the marronoid clade (excl. the Nearctic Amaurobiinae), as found in the ML analysis. Some minor disagreement between ML and BI trees was also found among the New Zealand Matachiinae, which is not considered to have an effect on the present comparative analysis.

The relationships between major taxa overlaps in large parts with the previous findings of Wheeler *et al*. [18], who used only six short genetic markers and a smaller taxon sampling for the Austral families (i.e. Desidae, Stiphidiidae, Cycloctenidae, Toxopidae). Our results show a strong need for the revision of the ‘marronoid' families, a problem that has been flagged by arachnologists for a long time [18]. Our phylogenetic results provide the first evidence on the placement of problematic taxa, that have been found extremely difficult to place into a family based on morphological characters alone. For instance, we found that the New Zealand ‘Amaurobiidae' and ‘Agelenidae' form a clade with Cycloctenidae, that the Australian amaurobiid genus *Daviesa* is a sister lineage of Porteriinae (Desidae), the genus *Toxopsoides* (currently doubtfully placed in Toxopidae) is a sister lineage of Amphinectinae *sensu stricto* (Desidae) and the genus *Wiltona* (former Tengellinae) falls into Stiphidiidae (all these relationships were highly supported with ubf-values greater than 95). Further, our data showed that the problematic genera *Aorangia* and *Cicurina* each form lineages outside currently defined families and confirmed that the water spider *Argyroneta* belongs to Dictynidae. The formal revision of the systematics of the marronoid group will be dealt with in a separate work, based on an enhanced taxon sampling and including morphological characters.

This first broader-scale insight into the phylogeny of the marronoid clade is highly relevant for the understanding of the remarkably dynamic phenotypic evolution of this group: it shows that taxa with divergent foraging modes (web builders versus cursorial), body shapes and sizes often group together.

### (b) Diversity and evolution of web-building behaviour, running speed and leg spination

We gathered ecological data for most studied species, including many original observations that represent the first descriptions of webs and foraging ecology for many of the studied species (electronic supplementary material, S7 and S8). This natural history data reveal an enormous diversity of web shapes and hunting styles throughout the marronoid clade of spiders.

The phylogenetic comparative analysis of foraging style indicated a highly dynamic evolution of web-building behaviour in the marronoid clade. Transitions between web-based and non-web-based foraging occurred repeatedly across our taxon sample, with slightly more web losses (13) than gains (10) if equal rates were assumed, and 30 web losses if web regain was suppressed. These results were independent of the position of Agelenidae.

Maximum running speed was lowest (5–8 body lengths per second, bl/s) in individuals of the cursorial spiders *Plectophanes* sp. and *Desis marina*, and the web builders *Paramatachia decorata* and *Taurongia* sp. 3 (a summary of all comparative data can be found in S8, and raw data in electronic supplementary material, S2 and S3). Running speed was highest (over 100 bl/s) in individuals of the cursorial spiders *Toxopsoides* sp. 9 (holding the record with 138 bl/s) and *Toxopsoides* sp. 10, as well as individuals of the web-building species *Procambridgea hunti* and *Pillara griswoldi*.

The phylogenetic mapping of running speed (bl/s) showed clear genus or clade-specific trends (figure 1). Notable trait differences between sister lineages were rarely associated with changes in foraging mode. For example, there were several switches in trends of running speed within Stiphidiidae, which (with the exception of *Wiltona*) are all web builders, and there were no major changes in sprint speed in Matachiinae following web losses.

Phylogenetic linear models did not indicate significant differences in running speed between web builders and cursorial hunters (mean difference = 2.03, 95% CI (−6.78, 11.14); *p* = 0.435; and *p* = 0.443, if Agelenidae was constrained at the tree base; figure 1 inset) nor between ecribellar and cribellar (mean difference = 6.70, 95% CI (−1.56, 14.64); *p* = 0.155; and *p* = 0.153, if Agelenidae was constrained at the tree base) and between ground-dwelling and above-ground-dwelling species (2.54, 95% CI (−4.88, 9.93), *p* = 0.192; and *p* = 0.193, if Agelenidae was constrained at the tree base; electronic supplementary material, S9).

The average spination index differed between web builders and cursorial spiders with the latter exhibiting, on average, more and/or longer spines per unit leg length (phylogenetic linear model, *p* = 0.034; and *p* = 0.033, if Agelenidae was constrained at the tree base), but the effect size was very small (0.244, 95% CI (0.062, 0.451)). Spination did not differ significantly between cribellar and ecribellar (0.141, 95% CI (−0.005, 0.285), *p* = 0.115; and *p* = 0.113, if Agelenidae was constrained at the tree base) nor between ground-dwelling and above-ground-dwelling species (−0.119, 95% CI (−0.276, 0.032), *p* = 0.288; and *p* = 0.282, if Agelenidae was constrained at the tree base; electronic supplementary material, S9). Running speed and spination index were not correlated (*p* = 0.335 for both topologies).

## 4. Discussion

Our results support the supplementation rather than the substitution hypothesis of extended phenotype evolution, with potential interactions and trade-offs between specific spider and web traits.

The evolution of running speed was poorly correlated with the use of webs as extended prey capture devices—both traits showed mosaic, independent evolutionary patterns. This indicates that the use of webs does not reduce the selective pressure on locomotory performance *per se*.

It is possible that an arms-race-like predator–prey interaction, where counter-strategies of some prey to reduce the efficiency of traps, maintains the selective pressure on speed. Many web-building marronoid spiders produce complex adhesive compound threads based on dry nanofibres, the so-called cribellar silk. It has been shown that some hair and scale-like surface features of the prey's cuticle highly reduce the stickiness of cribellar silk [49]. In addition, cribellar silk has been shown to interact with wax coatings on insect cuticles to form an adhesive bond [50], which simultaneously stiffens the threads, which may inadvertently help active prey to break free [51]. High sprint speed is advantageous in such situations in which the web's capacity to immobilize the prey is compromised, as the spider has to move fast to prevent the quick escape of the prey for successful prey capture. Larger webs, such as the sheet webs of many Agelenidae, boralline Stiphidiidae and porteriinae Desidae, may enhance the overall chance of prey interception, but require fast locomotion over longer distances in order to retrieve the prey before it can escape, as the spider typically rests in a funnel retreat at the edge of the sheet. Notably, many of such marronoid lineages that build large sheet webs and exhibit high running speeds (with the exception of Borallinae) have lost the ability to produce cribellar capture threads. By contrast, species that produce webs with thick and looped cribellar threads, such as *Paramatachia* spp. and *Neoramia* spp., that have the potential to immobilize prey longer [52], exhibited comparably slower running speed, which may indicate a trade-off between the investment in the cribellar spinning apparatus or the locomotory system. However, across the dataset running speed did not differ between cribellar and ecribellar spiders, showing that the lineage-specific evolutionary trajectories of locomotor performance cannot be explained with this trade-off alone.

Spiders are not only predators but also prey, and their locomotor performance may be under strong selection by predation. Webs may play an important role in predation defence by providing shelter [53], and hence we predicted similar effects on selection pressures acting upon locomotor performance as predicted for the web's function as an extended prey capture device. Yet, our results could not confirm that spiders sheltered from predation by webs have a reduced locomotor performance. Different types of webs might have different capacities to act as a shelter, especially in interaction with the microhabitat structure into which they are constructed and/or the type of predator [53,54]. Also, the process of web building and maintenance may expose spiders to predators, in a similar way as cursorial spiders are exposed during periods of active foraging, and therefore the capacity of webs to reduce fitness costs due to predation might be limited. Other anti-predator strategies that may render fast movement unnecessary (or even disruptive), such as crypsis or dragline dropping behaviour [54], have not been considered here, though they might play a role in some of the studied species, including both web-building and non-web-building species.

As locomotor performance is a composite trait affected by different morphological and physiological characters, it may indirectly be affected by adaptation to special microhabitats. For instance, *Paramatachia* spp. and *Plectophanes* sp. belong to the slowest species in our dataset. These species retreat into empty insect bore holes in wood or hollow twigs and accordingly have a slender body shape with short legs, which may be disadvantageous for locomotion. On the other hand, many species that typically retreat into narrow spaces in rotting logs or between the leaf bases of tussocks or rosettes showed high sprint speeds (e.g. species of *Pillara*, *Procambridgea* and *Toxops*). Among the fastest runners were the species with sideways tilted (laterigrade) legs (e.g. species of *Toxopsoides*, *Toxops*, *Cycloctenus* and *Manjala*)—a feature associated with flat bodies to squeeze into crevices but also permitting high manoeuvrability on flat substrates such as tree trunks [55]. Such species may often forage on exposed sites and take advantage of rapidly seeking shelter. Yet, not all super-performers had laterigrade legs—*Pillara* and *Procambridgea* were rapid runners even with a body shape and natural behaviour usually associated with pendulum mechanics [56] and foraging in non-exposed microhabitats in and under rotten logs.

As an example of hypothesized morphological adaptation towards prey capture, we analysed leg spination. Model results showed that cursorial spiders were more likely to have a greater number and longer spines on the front legs, but the difference in the global spination means between web builders and non-web builders was very small. Across the phylogeny there were multiple cases of web-building and non-web-building sister lineages, where the branch of the non-web-builder evolved greater front leg spination (e.g. *Storenosoma* versus *Tanganoides*; *Wiltona* versus Neoramia-group; *Daviesa* versus Porteriinae). However, in clades with the highest evolutionary dynamics in web use (such as Matachiinae and Amphinectinae), changes in foraging mode and the direction of spination evolution was seemingly not correlated. This could indicate that spination evolved gradually over longer time frames or that selection favours them only conditionally (e.g. depending on predatory strike behaviour; [13]).

Based on our observations and a previous study [13], the spines on the first pair of legs are primarily involved in prey capture, however, leg spines may also play some role in gaining a foothold on complex structured habitat surfaces [57]. If these spines are under selection for climbing locomotion, higher expression of spines would be expected in above-ground- than in ground-dwelling species—however, no such difference was found in our dataset.

Overall, these results do not suggest that extended prey capture devices substitute physiological and morphological adaptations of the body towards the functions of prey interception and retention. Trap-building strategies have comparably rarely evolved among predatory animals, and got repeatedly lost again subsequent to its evolution [5,8]. Our finding that extended prey capture devices serve as supplements rather than substitutions of body traits may indicate that the costs of trap building cannot be offset, and thus trap-building predation has elevated costs in comparison to active predation. It therefore follows that trap-based foraging is a costly strategy that pays off only in conjunction with special ecological and life-history strategies, e.g. if it results in a high prey yield and the predator is capable of using such enhanced resources for its rapid growth and increased reproduction [58]. Notably, trap-based predators often exhibit a high degree of plasticity in trap building with economized investments [9], which further supports that such predators operate at a comparably risky level. Traps and snares may also largely differ in their efficiency to capture prey, which may explain interspecific fitness differences [58]. More data are needed on the fitness costs and gains of different trap-based predatory strategies to pinpoint the niche within which they are evolutionarily successful.

## 5. Conclusion

Here, we have combined the first comprehensive phylogenomic analysis of the enigmatic marronoid clade of spiders with the large-scale comparative analysis of physiological, morphological and ecological traits. This enabled the first-time inference of how locomotor performance and morphological adaptations evolve on a deep time scale in animals that use extended phenotypes. Results show that the evolution of locomotor performance and front leg spination in spiders each exhibit complex dynamics that are not, or only poorly, correlated with the loss and gain of silken webs as extended prey capture and defensive devices. Extended phenotypes serving as substitutes for body traits may rather be the exception than the rule. Rather extended phenotypes serve as important supplementary assets, enhancing the functionalities of the body.

## Data Availability

Data available from the Dryad Digital Repository: https://doi.org/10.5061/dryad.0zpc8673w [59]. Supplementary material is available online [60].
